# Therapeutic vs. Recreational Use of Cocaine: Avoiding Diagnostic and Judicial Errors Through Interprofessional Collaboration—A Five-Case Report †

**DOI:** 10.3390/healthcare13182318

**Published:** 2025-09-16

**Authors:** Gaëlle Magliocco, Laurent Suppan, Tatjana Vujic, Cristian Palmiere, Aurélien Thomas, Silke Grabherr, Marc Augsburger

**Affiliations:** 1 University Center of Legal Medicine, Lausanne and Geneva University Hospitals, 1000 Lausanne, Switzerland; gaelle.magliocco@chuv.ch (G.M.);; 2 Unit of Forensic Toxicology and Chemistry, CURML, Lausanne and Geneva University Hospitals, 1000 Lausanne, Switzerland; 3 Division of Emergency Medicine, Department of Anesthesiology, Pharmacology, Intensive Care and Emergency Medicine, Geneva University Hospitals, 1205 Geneva, Switzerland; 4 Faculty Unit of Toxicology, CURML, Faculty of Biology and Medicine, University of Lausanne, 1000 Lausanne, Switzerland

**Keywords:** cocaine, nasal surgery, anesthesia, driving under the influence of drugs (DUID), toxicological analysis

## Abstract

**Background/Objectives**: Due to its potent local anesthetic and vasoconstrictive properties, cocaine is sometimes used in otolaryngologic surgical interventions. However, cocaine topical administration is not always adequately documented by practitioners, which can lead to serious legal consequences, particularly in the context of drug-impaired driving (DUID) investigations. This study retrospectively analyzes five road accident cases where cocaine was detected in biological samples after medical interventions. **Case descriptions**: Following pedestrian–car, or car–car accidents, five distinct patients aged between 30 and 84 years underwent maxillofacial surgery due to significant injuries. Given the severity of the accident and the circumstances, the police requested blood toxicological analysis to determine whether the patients were under the influence of psychoactive substances at the moment of the accidents. **Results**: The five cases described in this manuscript had blood cocaine concentrations exceeding the Swiss legal limit for drivers (15 µg/L). Since no information was initially provided about the medical use of cocaine after the crash, recreational use of cocaine was suspected. However, subsequent investigations confirmed that the cases involved medical administration. **Conclusions**: After sinonasal procedures involving the topical application of cocaine, patients may yield positive results on urine and blood drug tests, potentially resulting in serious legal repercussions, including the withdrawal of their driving license. Therefore, practitioners should thoroughly document the medical use of topical cocaine, particularly in DUID cases. These results also raise questions about the benefit–risk ratio of such use, considering that alternatives exist.

## 1. Introduction

Cocaine is a powerful central nervous system stimulant and local anesthetic [1]. It acts primarily by blocking the reuptake of dopamine, norepinephrine and serotonin into presynaptic neurons, thereby increasing the concentration of these monoamines in the synaptic cleft [2]. This mechanism underlies both its euphoric and sympathomimetic effects [1]. Cocaine is typically taken by users through smoking, nasal insufflation (snorting), or intravenous injection [3]. Although cocaine is absorbed more rapidly after being smoked or injected, nasal administration also allows fast absorption through the richly vascularized nasal mucosa, resulting in a rapid onset of action, with peak blood concentrations achieved within 30 to 60 min [4]. At neutral and basic pH, cocaine hydrolyses spontaneously into benzoylecgonine (BZE). Carboxylesterases also play an important role in cocaine metabolism, catalyzing the hydrolysis of cocaine to BZE and ecgonine methyl ester (EME). Butyrylcholinesterase is also involved in the metabolism of cocaine to EME. When ethanol is consumed with cocaine, another metabolite is formed, ethylcocaine (EC) [5].

Cocaine may affect a driver’s ability through different mechanisms, such as impaired reaction time, increased feelings of confidence and driving skills, increased risk-taking, decreased concentration and judgment, and increased aggressiveness [6]. In Europe, during the last decade, an increase in cocaine consumption was observed, which is now the second most used illicit drug, after cannabis [7]. As a consequence, a rise in the number of drivers suspected by the police to drive under the influence of this substance was observed. Cocaine consumption among drivers suspected of driving under the influence of drugs in Switzerland has also shown an overall upward trend over the last 30 years [8,9,10]. To deal more effectively with drugged drivers, several countries have enacted a zero-tolerance law (“per se” law) for illegal drugs such as cocaine [11,12]. Therefore, since 2005, a zero-tolerance approach was introduced into Swiss legislation, notably for cocaine. A technical cutoff was set out in the law at 15 µg/L in whole blood, with a measurement uncertainty of ±30% [13]. These limits apply, regardless of the behavioral or clinical assessment of the driver, meaning that any blood concentration exceeding these thresholds constitutes an offense, even in the absence of obvious signs of impaired driving ability. Regarding other countries, Gjerde et al. reviewed the legal limits for driving under the influence of illicit drugs, including cocaine, across different jurisdictions [14]. Legal blood concentration thresholds for cocaine typically range from 5 to 50 µg/L. For instance, New Zealand enforces a strict limit of 5 µg/L, whereas the Netherlands permits up to 50 µg/L when cocaine is detected in the absence of other substances.

In the context of maxillofacial surgery, a surgical modality is sometimes required following motor vehicle accidents, as cocaine can be used as a topical anesthetic and vasoconstrictor [15]. According to Lutfallah et al., a maximal safe dose of 200 mg, or 1.5 mg/kg to 3 mg/kg, of intranasal cocaine is typically used during surgery [16]. This medical application can potentially complicate toxicological interpretation, as the presence of cocaine or its metabolites in biological specimens may reflect either therapeutic administration or recreational consumption. The differentiation between these sources can be challenging, particularly in cases where comprehensive medical documentation is unavailable or when the timing between surgical intervention and specimen collection is not clearly established.

To illustrate this, we present five retrospective cases of road traffic accidents in which blood and urine samples were collected during hospitalization for routine toxicological analyses in our forensic toxicology and chemistry unit. They were specifically selected because their measured cocaine concentrations in blood and/or urine were inconsistent with recreational cocaine use. Information about the cases (i.e., age, date/hour of the event/sampling) was obtained from the police analysis order. For all these cases, we initially received no information regarding a potential use of topical intranasal cocaine as part of nasal interventions.

## 2. Analytical Workflow

For all cases, the general analytical strategy was similar. First of all, systematical toxicological analyses were performed on blood and urine (when available), using immunoassays and gas chromatography-mass spectrometry (GC-MS) on urine. To identify peaks, GC-MS results were compared to several databases, such as Maurer/Pfleger/Weber [17], Wiley, and the National Institute of Standards and Technology (NIST). Positive results were confirmed through quantification in biological samples using GC-MS, gas chromatography-tandem mass spectrometry (GC-MS/MS) or liquid chromatography-tandem mass spectrometry (LC-MS/MS) [8,10]. Alcohol was both qualified and quantified in blood using headspace gas chromatography coupled with flame ionization detector (HS-GC-FID). Biological samples were stored at −20 °C or below from the time of collection until analyses to minimize degradation and ensure the reliability of toxicological results.

## 3. Case Descriptions

### 3.1. Case I—Pedestrian–Car Accident

An 83-year-old man was admitted to the emergency room after he was hit by a car on a pedestrian crossing. He underwent several surgeries, including maxillofacial surgery. Because of the severity of the accident and the context, the police requested blood toxicological analysis of the pedestrian and the driver, to determine if they were under the influence of psychoactive substances at the moment of the accident. Blood and urine were obtained from the pedestrian 11 h 2 min and 11 h 4 min after the accident, respectively. Midazolam, ketamine and lidocaine, commonly used during medical interventions, were also detected in urine. Cocaine (16 and 1700 µg/L, respectively), BZE (370 and 560 µg/L, respectively) and EME (60 and 1200 µg/L, respectively) were detected in the blood and urine (Table 1).

### 3.2. Case 2—Pedestrian–Car Accident

An 84-year-old woman was admitted to the emergency room after she was hit by a car on a pedestrian crossing. She underwent several surgeries, including maxillofacial one. Because of the severity of the accident and the context, the police requested blood toxicological analysis of the pedestrian and the driver, to determine if they were under the influence of psychoactive substances at the moment of the accident. Blood from the pedestrian was obtained 1 h 25 min after the accident, and no urine sample was available. Only cocaine (230 µg/L), BZE (370 µg/L), and EME (42 µg/L) were detected in the blood (Table 1).

### 3.3. Case 3—Car–Car Accident

A 66-year-old man, who was passenger in a car involved in a collision with another vehicle, was admitted to the emergency room with serious injuries. He underwent several surgeries. He died 2 h 3 min after the accident. Because of the severity of the accident and the context, the police requested blood toxicological analysis of the drivers and passengers to determine if they were under the influence of psychoactive substances at the moment of the accident. Blood and urine from the passenger were obtained 1 h 30 min after the accident. Additional blood samples were collected 2 h after the accident and during the autopsy. Metabolites of bromazepam, lidocaine, and ibuprofen were detected in post-mortem urine. No cocaine or metabolites were detected in ante- and post-mortem urine samples. Cocaine (370 µg/L), BZE (200 µg/L) and bromazepam (97 µg/L, benzodiazepine) were detected in post-mortem blood. In blood samples collected at T0 + 01:30, no cocaine or metabolites were detected. In blood samples collected at T0 + 02:00. cocaine (1800 µg/L), BZE (460 µg/L), were detected (Table 1).

### 3.4. Case 4—Car Accident

A 30-year-old man involved in a car accident was admitted to the emergency room because of serious injuries. Because of the severity of the accident and the context, the police requested blood toxicological analysis of the driver to determine if he was under the influence of psychoactive substances at the moment of the accident. Blood and urine were obtained 4 h 5 min after the accident. Ethanol (1.32 g/kg), THC (2.0 µg/L), 11-OH-THC (<1.0 µg/L, active metabolite of THC), THC-COOH (38 µg/L, inactive metabolite of THC), midazolam (350 µg/L, benzodiazepine), hydroxyl-midazolam (20 µg/L, metabolite of midazolam), cocaine (29 µg/L), and BZE (<10 µg/L), lidocaine (local anesthetic), and fentanyl (opioid) were detected in the blood (Table 1). Neither cocaine, nor its metabolites were detected in the urine. Of note, midazolam, lidocaine and fentanyl are commonly used substances in medical interventions.

### 3.5. Case 5—Pedestrian–Car Accident

An 82-year-old man hit by a car was admitted to the emergency room because of trauma. He underwent several surgeries, including maxillofacial. Because of the severity of the accident and the context, the police requested blood toxicological analysis of the pedestrian and the driver to determine if they were under the influence of psychoactive substances at the moment of the accident. Blood and urine from the pedestrian were obtained 4 h 24 min and 4 h 29 min after the accident, respectively. Cocaine (32 and 190 µg/L, respectively), and BZE (45 and 120 µg/L, respectively) were detected in the blood and urine (Table 1).

## 4. Discussion

**Figure 1:**
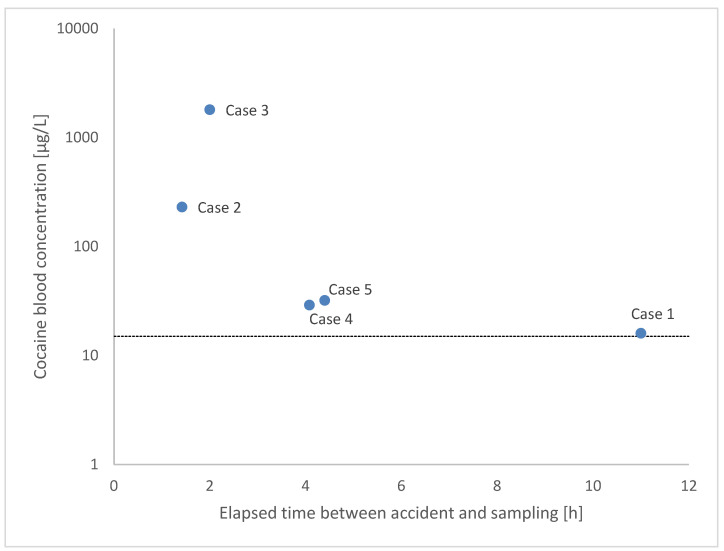
Concentration of cocaine in blood for the four cases. Dotted line: threshold limit of cocaine in blood as defined by Swiss legislation.

Swiss legislation sets a technical threshold limit of cocaine in blood at 15 µg/L ± 30% whilst driving, a blood concentration largely reached by the five cases described in this manuscript, as shown in Figure 1. Because no information was initially available concerning medical use of cocaine after the crash, recreational use of cocaine was suspected.

In these specific cases, the following elements caught our attention and led us to further investigations:

1. Three cases involved patients between 82 and 84 years old, whereas the mean age of cocaine consumers in Switzerland is around 34  ±  14 years in cases of driving under the influence of drugs (DUID) [8].
2. The context of hospitalization, which may be consistent with a potential therapeutic use of cocaine.
3. The detection of cocaine and its metabolites in blood, but not in urine (Case 3). This scenario suggests a very recent cocaine administration since the T_max_ of cocaine is approximately 0.43 ± 0.34 h and the cocaine metabolites, benzoylecgonine and ecgonine methyl ester, reach maximum urine concentrations within the 4 to 8 h collection period following topical intranasal administration [18,19].
4. Inconsistent results within multiple samplings (Case 3).

This research work raises three important concerns related to this procedure. The first important point concerns the need to provide clear documentation to subjects receiving medical treatments involving topical cocaine, such as hospital discharge summaries, in order to justify positive urine drug tests. Reichman et al. [20] showed in a study of 30 volunteers that cocaine used for medical purposes made a drug test positive for up to 3 days. In a recent prospective study involving 75 patients who received 80 mg of cocaine as a nasal spray during nasotracheal intubation, Larsen et al. [21] observed that cocaine was still detected in whole blood above the legal fixed limit of 0.01 mg/kg in 3% of the patients 24 h after the administration. It is important that patients are informed about the use of such an intranasal solution, including the fact that they may fail a drug-driving test for several days afterwards [22].

Secondly, it is essential for forensic toxicologists to be informed about the use of topical cocaine during medical practice to accurately interpret toxicological results, especially in the case where toxicological analyses are performed on samples collected shortly after a nasal surgery to search for the presence of drugs. Regarding forensic road traffic cases, personal therapeutic records are typically transmitted when the individual is conscious and able to provide it, which is often not the case in severe accidents. However, pharmacological treatments administered during hospitalization are almost never transmitted to our unit, mainly due to long-standing practices and the absence, until recently, of a need for such information. This lack of communication now represents a real limitation in forensic toxicological interpretation and should be re-evaluated. In a different context, Basilicata et al. have shown why an accurate medical history is crucial to avoid delays in toxicological diagnosis [23].

In rare cases, then, it is possible that a physician’s report of intranasal cocaine use during surgery could mask cocaine consumption in drug-addicted individuals, as it would be difficult for the toxicologist to distinguish between medical and recreational cocaine intake. In addition, cocaine is sometimes used as a topical anesthetic in ophthalmic surgery. To our knowledge, systemic absorption of this drug after ocular application has never been assessed and deserves further attention for the same reasons explained above [24].

Thirdly, while we are aware of the efficacy of intranasal cocaine solution as a vasoconstrictor and anesthetic agent, our second concern involves the safety issues associated with its use in medical practice. As stated in previous articles, during surgical procedures, cocaine may be used as an aerosol sprayed [20], as a 25% cocaine paste [25], or applied intranasally on a cotton pledget soaked in a 4–10% cocaine solution [26,27,28]. The systemic absorption of cocaine, and therefore the plasma concentrations, showed an important variability between studies since it depends on the formulation used, the contact time with the mucous membranes, and the amount of aerosol, paste or solution applied. As an example, in the recent study by McGrath et al. [18], 30 healthy volunteers received intranasal cocaine from a cotton pledget soaked in 4 mL of a 4% solution (equivalent to 160 mg of cocaine hydrochloride). The mean maximum observed plasma concentration (C_max_) was 37.0 ± 17.3 ng/mL. In some hospitals, cocaine is combined with epinephrine to decrease its absorption. Page et al. [27] showed that the mean peak plasma concentration of cocaine was 20.35 ng/mL (range 13–31 ng/mL) in twelve subjects who received topically Moffett’s solution containing 100 mg cocaine hydrochloride and 1 mg ephedrine. All these concentrations are consistent with those observed in cases 1, 4, and 5 of our study, whereas significantly higher concentrations were found in cases 2 and 3. While we are once again drawing attention to the risk of biasing the results of toxicological analyses in the context of road traffic, we should notice that some controversy exists among scientists regarding the benefit–risk balance of topical cocaine use. On the one hand, some argue about its very effective local properties of anesthesia and vasoconstriction [29], while others fear its potential toxicity [15]. Most of the reported complications were related to the cardiotoxic effects of cocaine, with tachycardia and hypertension being the most frequently recorded adverse events [29,30]. Although rare, serious adverse effects include, among others, seizure, death, and cardiac arrest [15]. According to McGrath et al. [31], these events were the result of cocaine administration either in patients with preexisting cardiovascular disease, or in association with other topical vasoconstrictors such as epinephrine. Storing cocaine in medical facilities can also lead to other types of complications, such as theft, cocaine addiction among medical staff, or administrative difficulties regarding storage [15,32]. In an emergency treatment setting, the patient’s medical history is often not known. In this context, the consequences of administering intranasal cocaine for local anesthesia to former cocaine addicts should also be considered. Other substances or combinations of substances, such as lidocaine or tetracaine with oxymetazoline, have proven to be effective alternatives to cocaine [32,33,34]. A recent research article also demonstrated the efficacy of topical tranexamic acid during endoscopic sinus surgery [35]. Although no official recommendations have been made, two recent reviews concluded that cocaine should be avoided when alternatives are available [16,36].

In 2019, a survey reported that 27% of Canadian otolaryngologists used cocaine for endoscopic sinus surgery [37]. In less recent research articles, other nations showed a higher prevalence (i.e., 64% in Australia, 68% in the United Kingdom, 50% in the United States) [15,30,38]. The prevalence of cocaine use during nasal surgery in Switzerland is not well documented and requires further investigation. A prospective study by Yeo et al. [22] conducted in the UK revealed that among 123 patients surveyed, 73% would prefer to be informed about cocaine use before surgery, and 83% expressed concerns about its illicit status (e.g., workplace or driving drug testing). Additionally, 34% of patients stated they would prefer a substitute for cocaine.

It is important to note that the results presented in this study reflect forensic procedures specific to Switzerland. National practices may vary with regard to the location of blood sampling, which may take place directly at the scene of the accident, or later in hospital. The categories of individuals from whom samples are collected may also vary between countries. For instance, in some countries, blood samples are not systematically taken from pedestrians involved in road accidents. Furthermore, the use of nasal cocaine in maxillofacial surgery is not permitted in all countries. Such differences can affect both the availability of toxicological data and its interpretation and should be considered when comparing results across different medico-legal systems.

## 5. Conclusions

In conclusion, the presence of cocaine or its metabolites in biological specimens may reflect either therapeutic administration or recreational consumption. Nasal administration of cocaine avoids the first-pass hepatic effect, allowing rapid systemic absorption of cocaine at significant and, presumably, non-risk-free blood levels during nasal surgery. It is popular among otolaryngologists because of its strong local anesthetic and vasoconstrictive properties. We recommend that the use of topical cocaine during nasal surgery should be further documented by practitioners, especially in the case of DUID. Indeed, toxicologists should be aware that such situations may arise when analyzing cocaine results, as patients may test positive for cocaine on urine and blood drug screens with legal consequences such as driving license withdrawal. It is also essential to inform patients about such use before surgery, when possible, to ensure their informed consent, and particularly after the procedure to allow them to take the necessary precautions.

## Figures and Tables

**Table 1:** Concentration of cocaine and metabolites in blood and urine.

Case	Sex	Age	Time Elapsed Between Accident and Blood Sampling	Blood (µg/L)				Time Elapsed Between Accident and Urine Sampling	Urine (µg/L)			
				Cocaine	BE	EME	EC		Cocaine	BE	EME	EC
#1 Traffic accident, pedestrian	Man	83	11 h 2 min	16	370	60	nd	11 h 4 min	1700	560	1200	nd
#2 Traffic accident, pedestrian	Woman	84	1 h 25 min	230	370	42	nd	(no urine sample)	-	-	-	-
#3 Traffic accident, car passenger	Man	66	1 h 30 min	nd	nd	-	nd	1 h 30 min	nd	nd	-	nd
			2 h	1800	460	-	nd	-	nd	nd	-	nd
			Autopsy	370	200	-	nd	-	nd	nd	-	nd
#4 Traffic accident, car driver	Man	30	4 h 5 min	29	<20	nd	nd	4 h 5 min	nd	nd	nd	nd
#5 Traffic accident, pedestrian	Man	82	4 h 24 min	32	45	<10	nd	4 h 29 min	190	120	nd	nd

Legend: BE: benzoylecgonine; EC: ethylcocaine; EME: ecgonine methyl ester; nd: non detected; -: not determined.

## Data Availability

The data presented in this study are available on request from the corresponding author. The data are not publicly available due to ethical reasons.
